# The differential diagnosis of parotid gland tumors with high-resolution ultrasound in otolaryngological practice

**DOI:** 10.1007/s00405-017-4636-2

**Published:** 2017-06-13

**Authors:** Anna Rzepakowska, Ewa Osuch-Wójcikiewicz, Maria Sobol, Raul Cruz, Ewelina Sielska-Badurek, Kazimierz Niemczyk

**Affiliations:** 1 Department of Otolaryngology, Warsaw Medical University, ul. Banacha 1a, 02-097 Warsaw, Poland; 2 Biophysics and Human Physiology Department, Warsaw Medical University, Warsaw, Poland; 3 Otolaryngology Department, Kaiser Permanente Medical Center, Oakland, USA

**Keywords:** Parotid gland tumor, Ultrasound, Pleomorphic adenoma, Monomorphic adenoma, Malignant parotid gland neoplasms

## Abstract

The aim of the study is to define the utility of ultrasound (US) in differentiating benign from malignant parotid tumors as well as pleomorphic adenomas (PA) from monomorphic adenoma (MA). Seventy-two consecutive parotid gland tumors were analysed with high-resolution ultrasonography (12 MHz) with color Doppler imagining. The histopathological diagnosis was confirmed after parotidectomy for each lesion. The sensitivity, specificity, accuracy, positive predictive value (PPV), and negative predictive value (NPV) for the US were established. Receiver operating characteristic curves were constructed to determine the predictive values of echogenicity, heterogeneity, and vascularity on color Doppler. Area under the curve (AUC) was calculated for each parameter considered. The analysed material included 27 MA, 26 PA, 1 basal cell adenoma, 8 inflammatory conditions, and 10 malignant neoplasms. The sensitivity, specificity, and accuracy of US in differentiation of malignant from benign lesions in the parotid gland were 60, 95.2, and 90.3%, respectively. The predictive values were: PPV 66.8% and NPV 93.6%. Differentiating diagnoses between PA and MA with US resulted in a sensitivity of 61.5%, specificity of 81.5%, and accuracy of 73.1%. The predictive values were: PPV 50% and NPV 68.8%, respectively. For distinguishing malignant from benign tumors, the highest AUC values noted were for heterogeneity and vascularization (0.8 and 0.743, respectively). The AUC values were the highest for hypoechogenicity and vascularization in separating PA from MA (0.718 and 0.685, respectively).

## Introduction

Overall 80% of parotid gland tumors are benign and are located in the superficial part of the gland. The most common neoplasm is pleomorphic adenoma (PA), also referred to as benign mixed tumor, representing about 60% of parotid gland tumors. The second commonest is monomorphic adenoma (MA), also known as papillary cystadenoma lymphomatosum, adenolymphoma, or Warthin’s tumor. Although PA is a benign tumor, it can relapse with recurrence rates of up to 6.8% [1]. In addition, PA have the potential for malignant transformation in 5–9.8% of cases [1]. These factors have led to recommendations against enucleation—these tumors should be resected with a cuff of surrounding normal glandular tissue, but preserving the facial nerve [2]. In contradistinction, MA are less aggressive lesions arising from remnant lymphoid ducts with little tendency to recur. Potentially, they could be resected with a less aggressive surgical procedure.

Even more crucial, the distinct between benign versus malignant nature of a parotid gland tumor is important in determining surgical recommendations. Benign lesions can be resected with some form of subtotal parotidectomy, while malignant lesions typically require total parotidectomies with the possibility of facial nerve resection. Since resected nerve is best reconstructed at the same stage, this can lead to a considerably more complex and time-consuming surgical procedure. Although there are some clinical clues of malignancy—rapid growth, skin fixation, ulceration, facial nerve palsy, pain, or cervical node metastasis—only 30% of malignant parotid gland cancers present with these features [3, 4]. The majority will be undistinguishable from benign tumors on presentation. Furthermore, the diverse histopathology of malignant salivary gland neoplasms presents as a broad spectrum of tumor morphology from the cystic appearance of low-grade mucoepidermoid carcinoma to the highly infiltrative character of follicular lymphoma [5].

Ideally, diagnostic investigations of parotid gland tumors would not only define the extent of disease, but also provide reliable information about the histopathological type of the tumor to aid in patient counseling and surgical planning. In modern clinical practice, high-resolution US examination is commonly used for the assessment of major salivary gland pathologies. The method is readily available, cost effective, avoids X-ray exposure, and increasingly performed by otolaryngologists. US with color Doppler allows the identification of even small pathologies within the parotid gland tissue with the assessment of perfusion pattern as well. However, there are a few limitations of this technology. The deep part of parotid gland cannot be thoroughly examined. The resolution of soft tissues is poorer than in CT or MRI [5]. Finally, the test’s quality depends highly on the investigator’s technical experience and interpretative skills [6].

The aim of our study was to evaluate the utility of US in the differentiation of various parotid gland neoplasms in a manner that might impact surgical planning and decision-making.

## Materials and methods

**Fig. 1:**
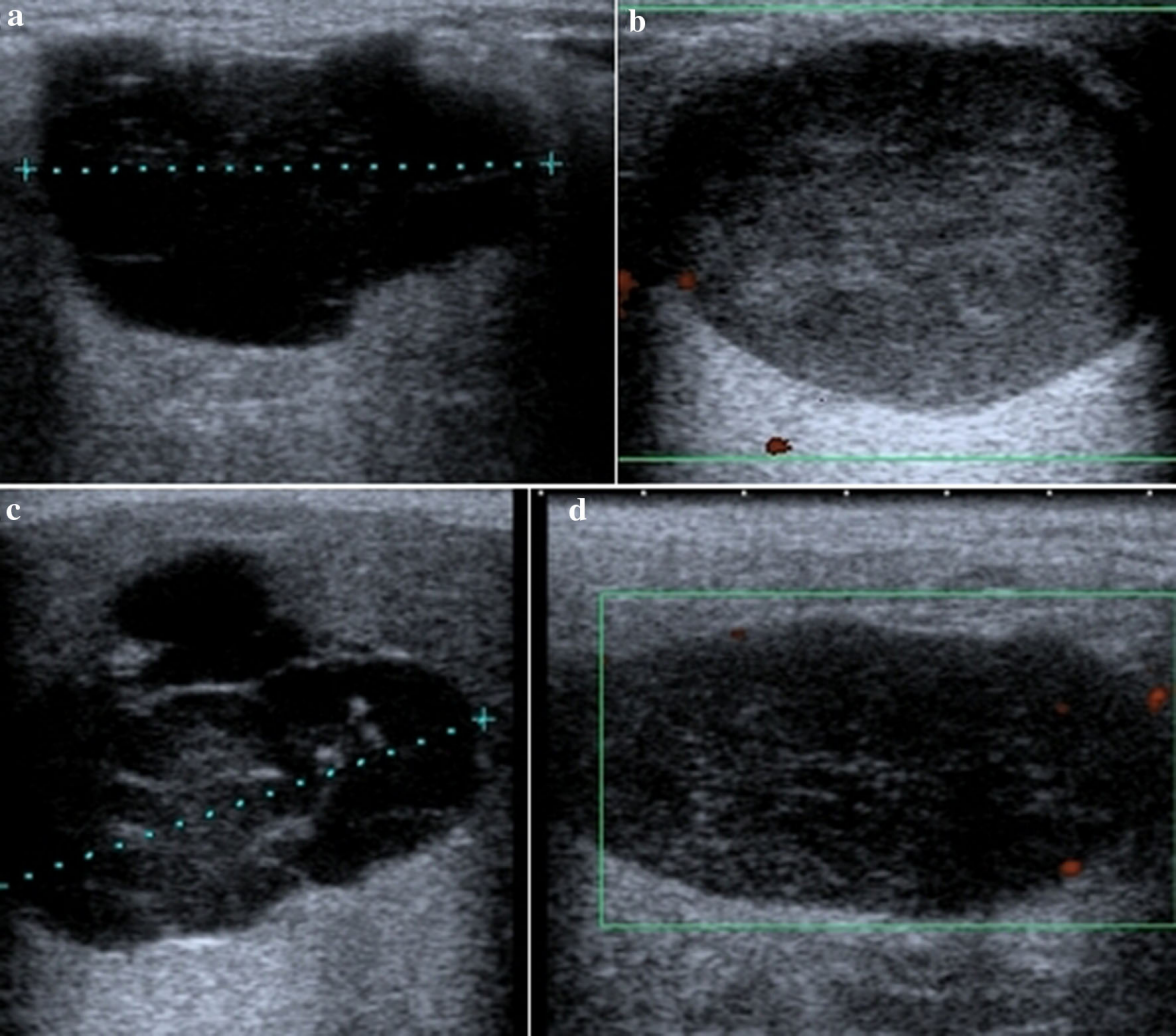
Examples of criteria for the description of the echogenicity (slightly hypoechoic, highly hypoechoic) and the homogeneity (slightly heterogenous, highly heterogenous) of the parotid gland tumors: **a** highly hypoechogenic, slightly heterogenic, **b** slightly hypoechogenic, slightly heterogenic, **c** highly hypoechogenic, highly heterogenic, and **d** slightly hypoechogenic, highly heterogenic

Ethical approval for this study was obtained from the Research Ethics Committee of the Warsaw Medical University. All patients gave their informed consent. During a period of 12 months, 72 patients (mean age 57.6 years, age range 20–83, 44 women, and 28 men), with parotid gland tumors, who had been admitted to our department for surgical treatment, were prospectively entered into the study. The exclusion criteria were: the presence of evident clinical features of malignant parotid gland tumor (skin infiltration and facial nerve paresis) and a history of radiation in parotid gland area. The US examination was performed by one otolaryngologist with over 5 years of experience in ultrasonography of the head and neck regions with a Toshiba Xario™ SSA-660A, V9.00 (Toshiba Medical Systems Corporation, Shimoishigami, Japan) 12 MHz linear array. The US examination was performed without knowledge of any prior US, fine needle aspiration biopsy (FNAB), or computed tomography (CT) results. The following characteristics were recorded: the size of the tumor (at least two dimensions), shape (regular, e.g., round or oval, irregular, e.g., polycyclic, lobular), and tumor margins (well-defined, poorly defined). The echogenicity (slightly hypoechoic, highly hypoechoic) and homogeneity (homogeneous, slightly heterogenous, highly heterogenous) of the tumor were established (Fig. 1). Based on color Doppler imaging, the vascular pattern within the tumor was defined (no or poor vessels, moderate vascularization, and high vascularization). If two or more intraparotid lymph nodes larger than 5 mm were noted, this was included as another defining characteristic. Using the American Joint Committee on Cancer neck node staging system, neck nodes from all five neck regions were evaluated in each patient for size, morphological structure, and vascular pattern. Synchronous tumors of the opposite parotid gland were noted.

**Fig. 2:**
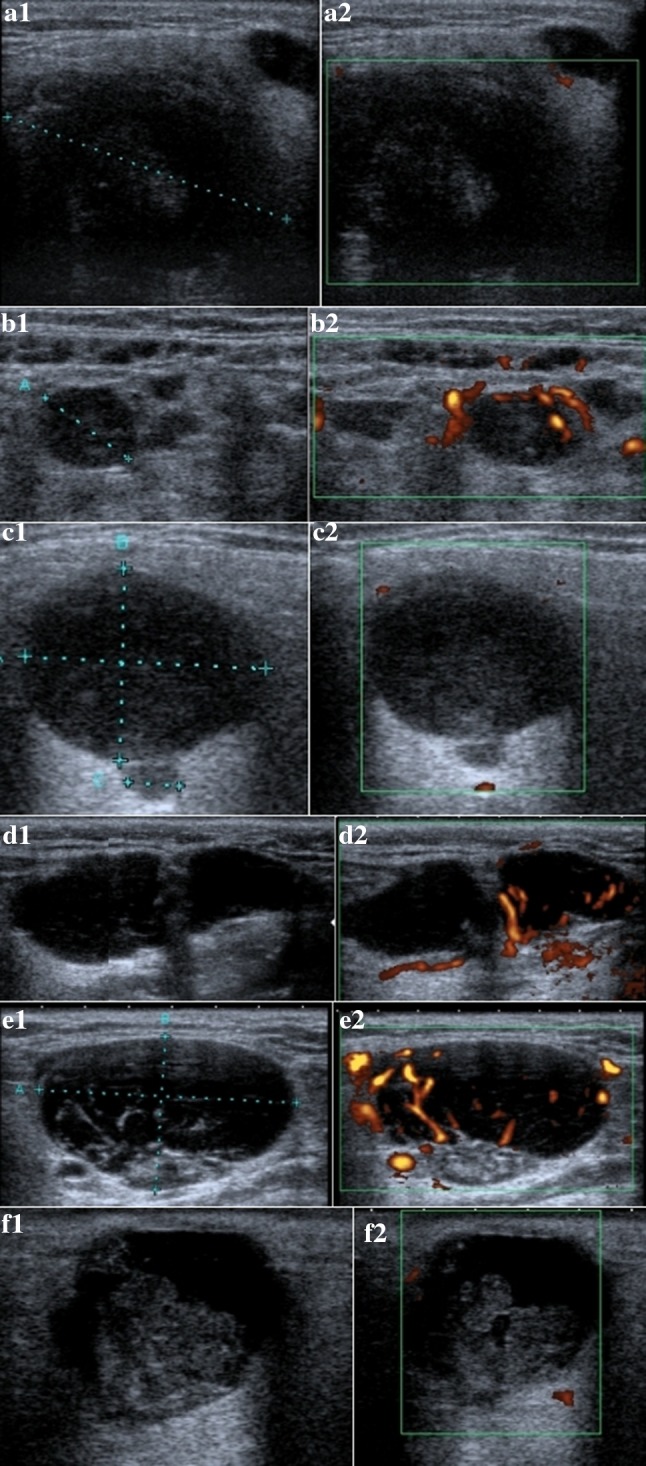
US of malignant parotid glands tumors: SCC—poor defined margins, highly hypoechogenic, slightly heterogenic, and poor vascularity pattern (**a1**, **a2**); MALT lymphoma—with regular, round shape, well-defined margins, slightly hypoechogenic, slightly heterogenic, and high vascularization (**b1**, **b2**); acinic cell carcinoma with regular, oval shape, well-defined margins, slightly hypoechogenic, slightly heterogenic, and no vascularization (**c1**, **c2**); follicular lymphoma with irregular, well-defined margins, highly hypoechogenic, slightly heterogenic, and high vascularization (**d1**, **d2**); mucoepidermoid carcinoma with regular, oval shape, well-defined margins, highly hypoechogenic, highly heterogenic, and high vascularization (**e1**, **e2**); adenoid cystic carcinoma with irregular shape, well-defined margins, highly hypoechogenic, highly heterogenic, and poor vascularization (**f1**, **f2**)

**Fig. 3:**
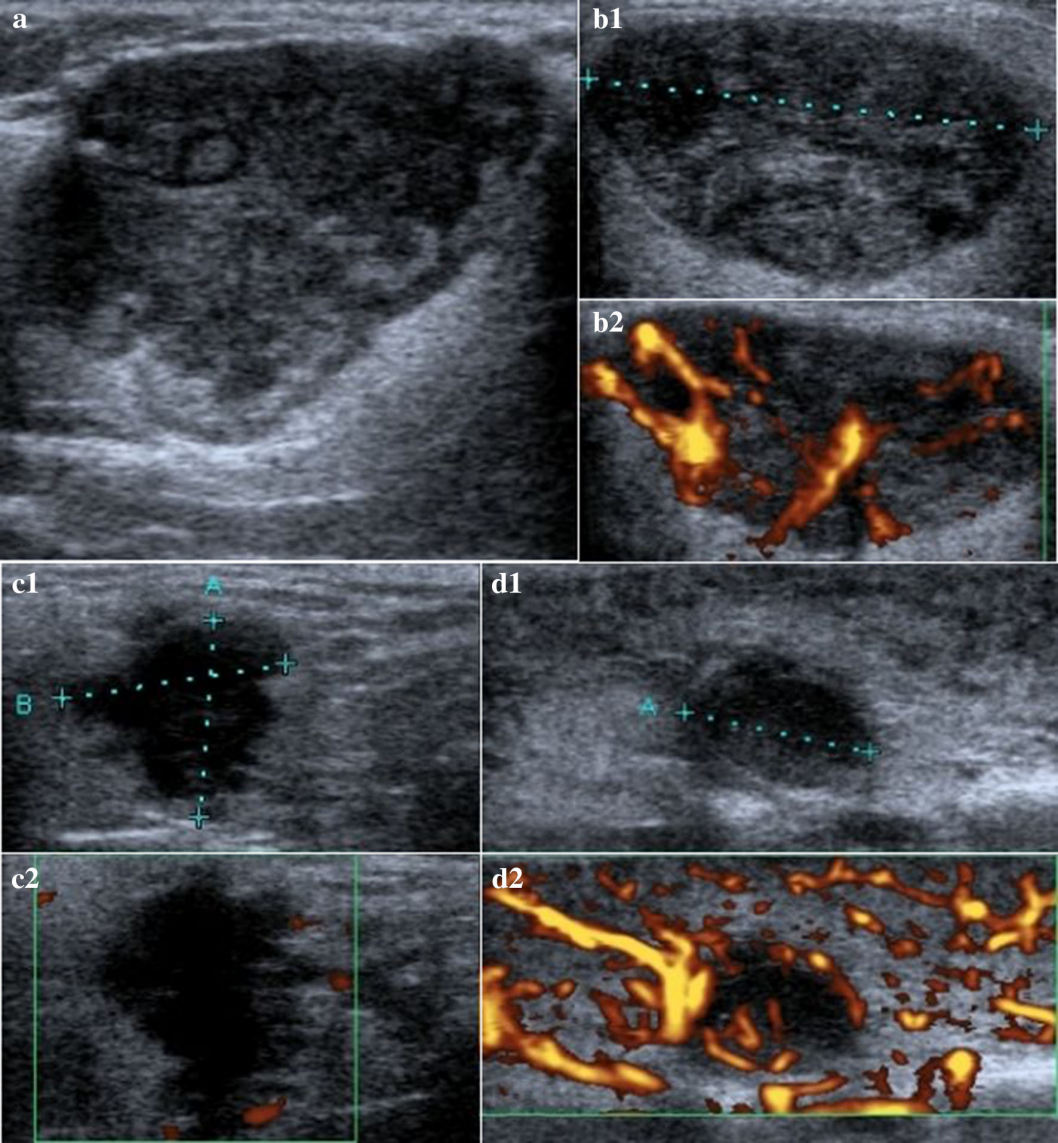
US of: PA with irregular shape, well-defined margins, slightly hypoechogenic, slightly heterogenic, and no vascularity (**a**); MA with regular, oval shape, well-defined margins, slightly hypoechogenic, slightly heterogenic, and high vascularization (**b1**, **b2**); adenocarcinoma with irregular shape, poor-defined margins, highly hypoechogenic, slightly heterogenic, and poor vascularity pattern (**c1**, **c2**); Sarcoidosis with regular, poor-defined margins, highly hypoechogenic, slightly heterogenic, and high vascularization (**d**1, **d2**)

**Fig. 4:**
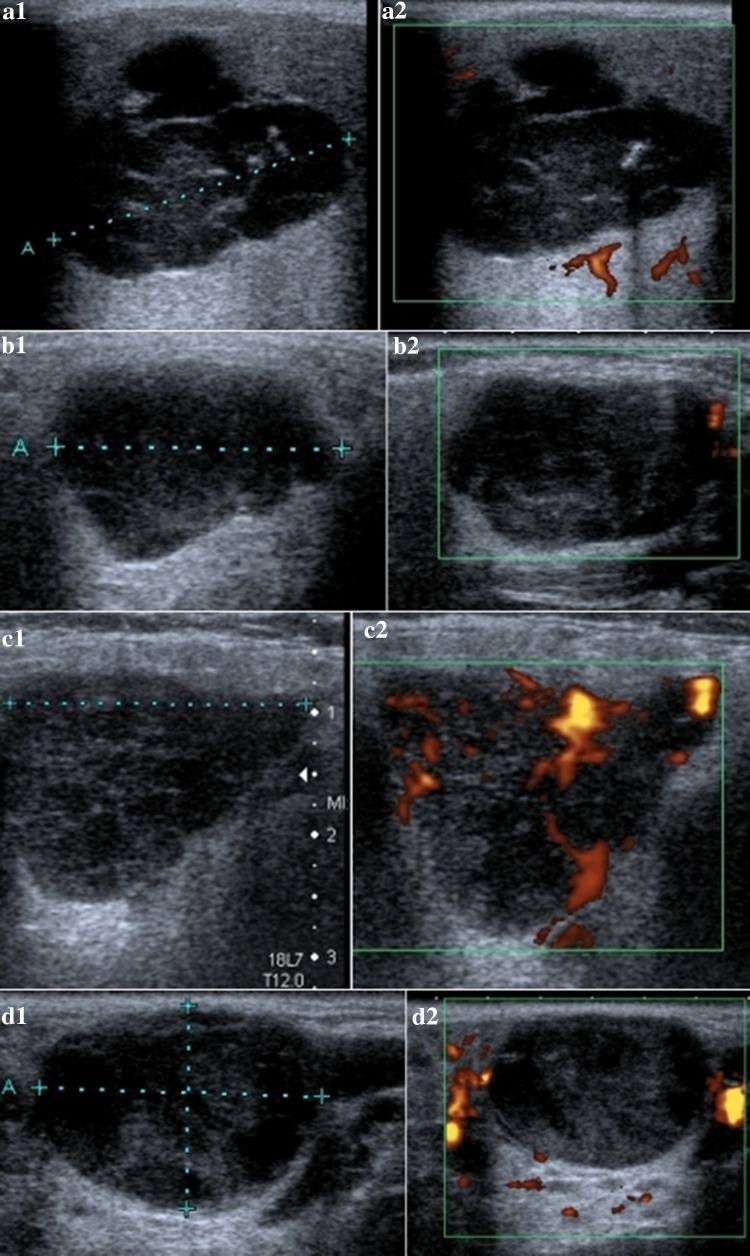
US of different pleomorphic adenomas: **a1**, **a2** polycyclic shape, highly hypoechogenic, highly heterogenic, and no vascularization; **b1**, **b2** polycyclic shape, slightly hypoechogenic, slightly heterogenic, no vascularization, and adenolymphomas; **c1**, **c2** polycyclic shape, slightly hypoechogenic, slightly heterogenic, and high vascularization; and **d1**, **d2** oval shape, highly hypoechogenic, highly heterogenic, and no vascularization

On the basis of the US examination, the investigator determined if the tumor was benign or malignant. The criteria of irregular shape, poor defined margins, heterogenous echogenicity, and increased vascular pattern were considered predictors of a malignant tumor (Fig. 2). If a benign tumor was diagnosed, it was decided whether it had the US morphology of PA or MA. PA were defined by the following characteristics: polycyclic or lobular shape, slightly heterogenous pattern, and poor vascularity). MA were predicted based on regular shape, highly heterogenous pattern, presence of enlarged intraparotid lymph nodes, and/or moderate or high vascularization (Figs. 3, 4).

The histopathological results obtained from the analysis of the surgical specimen were compared with US results including the diagnosis, tumor size and intraparotid, and cervical lymph node involvement. Statistical analysis was performed using the Statistica12 package StatSoft, Dell Statistica Partner. Categorical variables were summarized through the calculation of frequency. Continuous variables were summarized using descriptive statistics (mean, standard deviation, median, and range). Analysis of contingency table was done to calculate the sensitivity, specificity, positive predictive value (PPV), negative predictive value (NPV), and the accuracy. The Youden Index was calculated to evaluate the diagnostic power. Multivariate logistic regression analysis was used to construct a classification model to determine the variables statistically significant for patients with benign and malignant tumors and with PA and MA. The receiver operating characteristic (ROC) curves were constructed to determine the predictive values of echogenicity, homogeneity, and color Doppler. The performance of the model for classification was assessed by identifying the cut-off point of the predicted probability that yielded the largest sum of sensitivity and specificity. Moreover, area under the curve (AUC) was calculated for each considered parameter.

## Results

Based on the histopathological results, PA and MA were the two most numerous parotid gland tumors—26 and 27 cases, respectively. There was only one case of a basal cell adenoma. Malignant neoplasms were diagnosed in ten patients and the remaining eight cases were related to inflammatory conditions. The histopathological diagnoses are summarized in Table 1. Men with malignant tumors were the oldest group (mean age 68.5 years). The mean age of patients with PA was lower compared to patients with MA (51.5 and 63.7 years, respectively), especially in male patients (44.5 and 62.3 years, respectively). The relationships of mean age, gender, and histopathological results are presented in Table 2.

**Table 1:** Histopathological types of the pathology within 72 analysed parotid gland tumors

Histopathological results	Number (%)
Monomorphic adenoma (MA)	27 (37.5)
Pleomorphic adenoma (PA)	26 (36.1)
Basal cell adenoma	1 (1.4)
Inflammatory conditions	8 (11)
Chronic inflammatory state	4 (5.6)
Abscess	1 (1.4)
Sarcoidosis	1 (1.4)
Cystic degeneration	2 (2.8)
Malignant neoplasms	10 (13.8)
Adenoid cystic carcinoma	1 (1.4)
Acinic cell carcinoma	1 (1.4)
Adenocarcinoma	1 (1.4)
Follicular lymphoma	1 (1.4)
MALT lymphoma	1 (1.4)
Mucoepidermoid carcinoma	2 (2.8)
Salivary duct carcinoma	1 (1.4)
Squamous cell carcinoma (SCC)	2 (2.8)
Total	72 (100)

**Table 2:** Relationship of the mean age, gender, and the histopathological results of analysed patients with different parotid gland tumors

	Mean age according to histopathological results (years) ± SD					
	Monomorphic adenoma	Pleomorphic adenoma	Basal cell adenoma	Inflammatory conditions	Malignant neoplasms	All patients
Female	64.4 ± 8.0	52.7 ± 14.4	69	52.3 ± 19.5	53.5 ± 16.0	56.1 ± 14.4
Male	63.2 ± 9.0	44.5 ± 17.9		39 ± 12.7	68.5 ± 16.0	59.9 ± 15.1
Total	63.7 ± 8.5	51.5 ± 14.9	69	49 ± 18.2	62.5 ± 17.3	57.5 ± 14.7

Taking into account the sonomorphologic features, six out of ten malignant tumors were classified correctly by US. In four cases (mucoepidermoid carcinoma, adenoid cystic carcinoma, acinic cell carcinoma, and SCC), a malignant tumor was falsely classified as a benign one. In 59 (82%) patients, US confirmed the benign nature of the tumor. A false diagnosis of malignancy was predicted in three cases (two cases of chronic inflammatory changes and one case of parotid sarcoidosis). The sensitivity, specificity, and accuracy of US in differentiation of malignant versus benign lesion of the parotid gland were 60, 95.2, and 90.3%, respectively. The predictive values were calculated as: PPV 66.8% and NPV 93.6%. The Youden Index was 0.55.

US correctly diagnosed PA in 16 out of 26 cases and MA in 22 out of 27 cases. A false-positive diagnosis of PA was made in five cases and a false negative in ten cases. The ability of US to differentiate between PA and MA resulted in a calculated sensitivity of 61.5%, specificity of 81.5%, and accuracy of 73.1%. The resulting predictive values were: PPV 50% and NPV 68.8%. The Youden Index was 0.42.

A detailed analysis of US and histopathological criteria in different parotid gland pathologies is presented in Table 3. Malignant neoplasms and inflammatory conditions’ mean tumor size were slightly larger according to the US than on the histopathological results. In comparing mean tumor size as measured by US and histopathologic assessment, US was most accurate in measuring MA tumors. The mean size of PA was slightly underestimated with US.

**Table 3:** Comparison of US and histopathological (HP) criteria in different parotid gland pathologies

Analysed value or feature of tumor	Mean value or number (%) according to histopathological type of tumor					
	Monomorphic adenoma	Pleomorphic adenoma	Basal cell adenoma	Inflammatory conditions	Malignant neoplasms	All patients
Mean size in US (mm)	19.7 × 19.7	20.5 × 19.7	11 × 10	15.1 × 17	26 × 15.4	20.2 × 18.7
Mean size in HP exam (mm)	19.7 × 21.4	22.7 × 21.8	9 × 9	10.5 × 13	19.8 × 21.3	19.8 × 20.6
Shape (US)						
Regular (oval, rounded)	21 (78)	14 (54)	1 (100)	3 (37.5)	5 (50)	46 (61)
Irregular	6 (22)	12 (46)	0 (0)	5 (62.5)	5 (50)	28 (39)
Margin definition (US)						
Well defined	27 (100)	26 (100)	1 (100)	6 (75)	7 (70)	67 (93)
Poor defined	0 (0)	0 (0)	0 (0)	2 (25)	3 (30)	5 (7)
Echogenicity (US)						
Slightly hypoechogenic	12 (44)	22 (85)	1 (100)	2 (25)	4 (40)	41 (57)
Highly hypoechogenic	15 (55)	4 (15)	0 (0)	6 (75)	6 (60)	31 (43)
Homogeneity (US)						
Homogeneous	0 (0)	0 (0)	0 (0)	1 (12.5)	0 (0)	1 (1)
Slightly heterogeneous	22 (81)	23 (88)	1 (100)	3 (37.5)	4 (40)	53 (74)
Highly heterogeneous	5 (19)	3 (12)	0 (0)	4 (50)	6 (60)	18 (25)
Intraparotid lymph nodes (US)						
Not detected	14 (52)	21 (81)	0 (0)	3 (37.5)	7 (70)	45 (62.5)
Present	13 (48)	5 (19)	1 (100)	5 (62.5)	3 (30)	27 (37.5)
Regional lymph nodes (US)						
Not detected	23 (85)	21 (81)	1 (100)	5 (62.5)	7 (70)	57
Present	4 (15)	5 (19)	0 (0)	3 (37.5)	3 (30)	15
Intraparotid lymph nodes on HP exam						
None detected	22 (81)	22 (85)	1 (100)	5 (62.5)	8 (80)	58 (81)
Present	5 (19)	4 (15)	0 (0)	3 (37.5)	2 (20)	14 (19)
Vascularity on color Doppler						
No/poor	13 (48)	21 (81)	1 (100)	5 (62.5)	5 (50)	45 (62.5)
Moderate	11	5 (19)	0	2 (25)	1 (10)	19 (26.5)
High	3	0	0	1 (12.5)	4 (40)	8 (11)
Tumor in second parotid gland						
No	22 (81)	26 (100)	1 (100)	7 (77.5)	9 (90)	65 (90)
Yes	5 (19)	0	0	1 (12.5)	1 (10)	7 (10)
Total number of tumors	27 (100)	26 (100)	1 (100)	8 (100)	10 (100)	72 (100)

The majority of MA (78%) had a regular shape. Alternatively, PA and malignant neoplasms were in over 50% cases irregular.

The margins were well-defined in all benign parotid gland tumors and surprisingly only 30% of malignant neoplasms had poor margin definition.

Considering the echogenicity of tumors, PA were typically (85%) slightly hypoechoic, as opposed to MA and malignancies that were highly hypoechoic in more than 50% of cases.

Benign tumors presented in over 80% of cases as slightly heterogenous and malignant neoplasms in 60% were highly heterogenous.

In regard to color Doppler vascularity, MA and malignancies had the highest percentage of moderate or high vascularity (52 and 50%, respectively), while 81% of PA had no or poor vascularization.

Enlarged intraparotid lymph nodes were detected on US in 48% of MA; however, in only 19% of cases was there histopathological confirmation of reactive intraparotid lymphadenopathy.

We noted cervical lymphadenopathy most commonly in inflammatory conditions (37.5%) and malignant neoplasms (30%).

Finally, a synchronous tumor of the contralateral parotid gland was detected in 5 (19%) patients with MA.

**Fig. 5:**
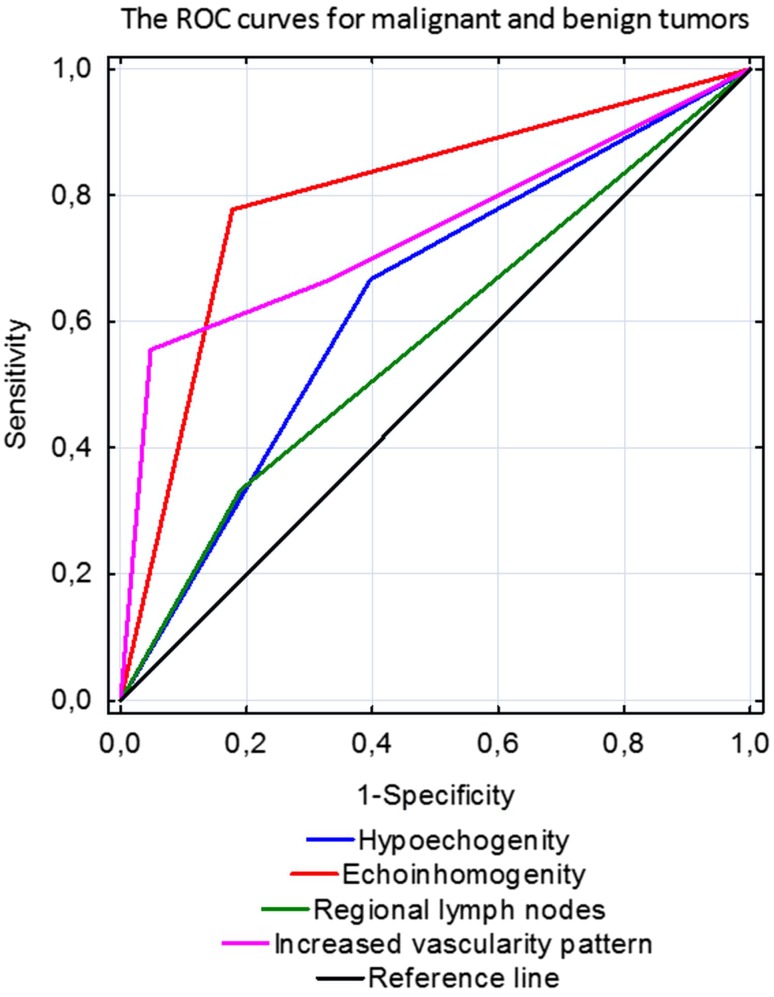
Receiver operating characteristic curves for malignant and benign parotid tumors

**Fig. 6:**
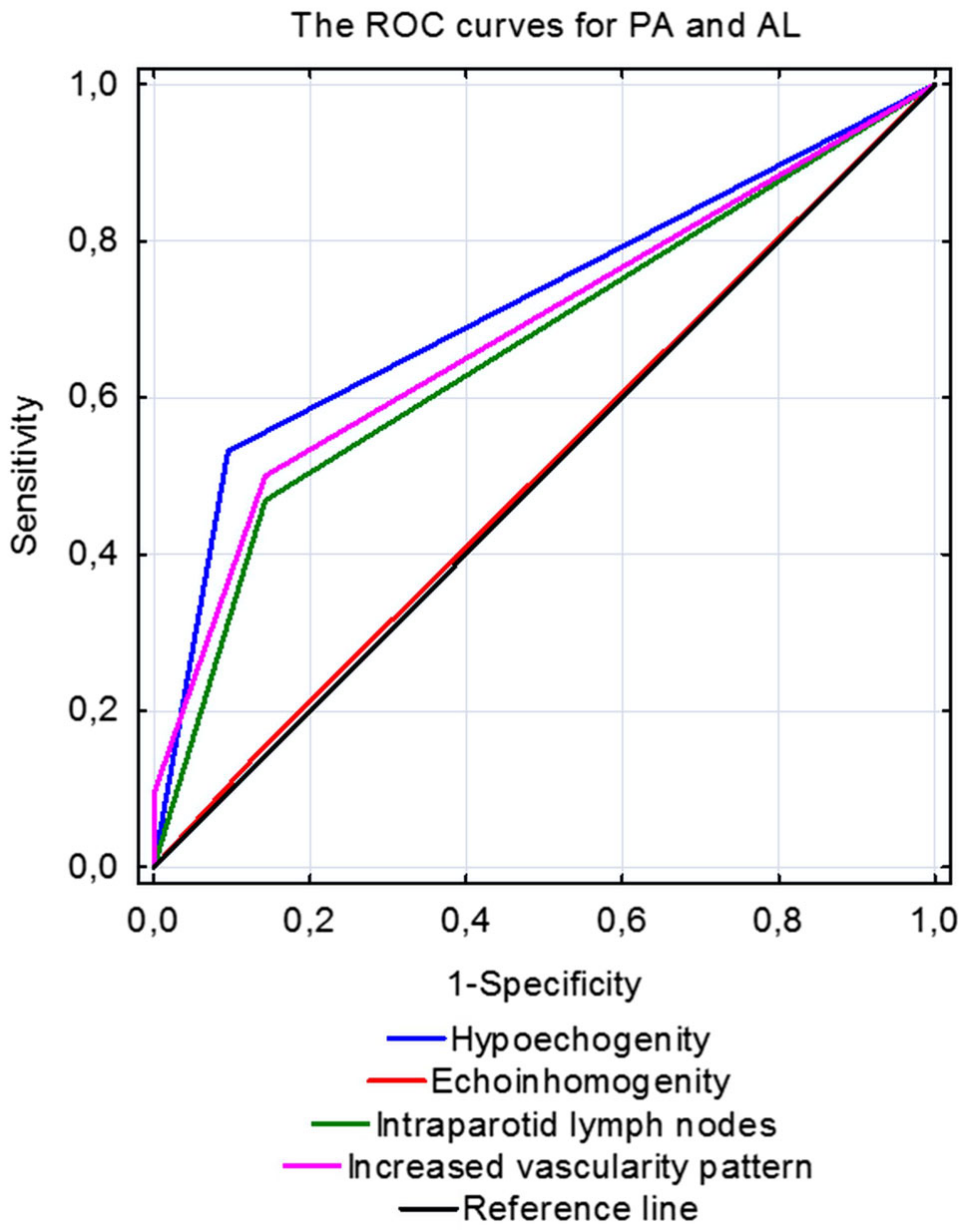
Receiver operating characteristic curves for pleomorphic adenomas/adenolymphomas of parotid gland

A classification model was constructed using multivariate logistic regression analysis to determine, which features of US examination are statistically significant for differentiation between malignant and benign tumors, as well as between PA and MA neoplasms. The results for each considered parameter are presented as the ROC curves for the determination of predictive value of hypoechogenicity, heterogeneity, increased vascular pattern on color Doppler, and enlarged intraparotid and regional lymph nodes in establishing the US diagnosis (Fig. 5 for malignant versus benign neoplasm and Fig. 6 for PA versus MA). The analysis of ROC curves showed that area under the curve for heterogeneity is 0.8 for patients with benign and malignant tumor while only 0.507 for PA and MA. This indicates that in the case of patients with benign and malignant tumors, heterogeneity assessment improved our ability to distinguish between the two groups. Our results indicate that for US differentiation between PA and MA, the most reliable parameters are hypoechogenicity and vascular pattern on color Doppler (AUC 0.718 and 0.685, respectively). Otherwise, for distinguishing malignant from benign tumor, the most essential are heterogeneity and vascularization (AUC 0.8 and 0.743, respectively). The calculated values for area under the curve (AUC) for each variable are summarized in Table 4.

**Table 4:** Calculated values of area under the curve (AUC) of ultrasonographic features in differential diagnosis of pleomorphic adenomas and monomorphic adenomas and malignant with benign parotid tumors

Parameter	AUC (area under the curve) value	
	PA/MA	Malignant/benign tumor
Hypoechogenicity	0.718	0.635
Heterogeneity	0.507	0.8
Increased vascularity on color Doppler	0.685	0.743
Regional lymph nodes	0.483	0.571
Intraparotid lymph nodes	0.663	0.540

## Discussion

Salivary gland tumors are responsible only for 3% of head and neck neoplasms. However, 80% of these tumors occur in the parotid gland. The gold standard for their treatment remains surgical resection. However, the topography of facial nerve course within the parotid gland tissue and the risk of postoperative facial paresis make parotid gland surgery challenging. The large variety of parotid gland tumor types and relatively rare occurrence can make their preoperative diagnosis difficult on clinical and radiologic grounds. As head and neck surgeons, preoperative diagnostic information can help us plan an adequate surgery anticipating the need for adequate margins and the potential for facial nerve reconstruction in the case of malignancy and a less radical procedure sparing the facial nerve in the face of a benign tumor. US as an increasingly accepted diagnostic tool has the potential to yield additional preoperative diagnostic information. This study attempts to identify sonographic features that would maximize the useful diagnostic information obtained from these studies.

The first challenge for US diagnosis of parotid gland tumors is differentiation of the clinically “silent” malignant tumor from benign growths. Following other authors’ guidelines, we assumed that irregular shape, poorly defined margins, heterogenic and hypoechoic structure, high vascularity, and regional lymphadenopathy are likely criteria for malignancy [5, 7–12]. Considering these potential factors in our study resulted in a sensitivity of 60%, specificity of 95.2%, and accuracy of 90.3% in differentiating benign from malignant parotid gland lesions. Other investigators have presented results of sensitivity ranging from 46.2 to 84%, specificity 88–98% and accuracy 57–96% [5, 8, 11, 13–15]. The four malignant tumors that were incorrectly diagnosed in our series were various pathologies—squamous cell carcinoma, acinic cell carcinoma, adenoid cystic carcinoma, and mucoepidermoid carcinoma. In three false-negative diagnostic cases, the tumor size was less than 15 mm; however, the SCC was a large tumor of 35 mm, but with the sonomorphological features of well-defined margins and poor vascularization. Bozzato et al. in his US study found that all their false-negative diagnoses of malignancy were in tumors less than 25 mm [6]. In our study, we expected that the sensitivity and specificity of US might change significantly with tumor size (the larger mass, the higher sensitivity, and specificity); however, we failed to prove it. Following other authors observations, we also believe that margin definition and echogenicity are not the reliable criteria for the differential diagnosis of malignant and benign lesions [6, 14, 16]. Bozzato et al. confirmed that nearly, 50% of their malignant neoplasms had well-defined margins [6]. Interestingly, in our study, all the three cases of false-positive diagnosis of malignancy involved inflammatory conditions, including very rare case of sarcoidosis. This was possible due to the highly echoinhomogenous texture of parotid gland without clear margins and relatively high vascularity of these inflammatory changes. Most inflammatory pathologies involve the whole parotid gland, but they can rarely take the form of focal involvement, misleading the clinician [5, 12]. Both in our study and by others researchers, the echoinhomogenicity and the increased vascularity pattern were identified as the most reliable features on US for the assessment of malignant character of the lesion [6, 17]. However, Bozzato et al. failed to find a characteristic perfusion pattern for malignancy within the parotid gland [6]. He felt that this was a result of small sample size and the histological variability of the few malignancies.

The second US diagnostic differentiation we looked at was between PA and MA. In our study, we calculated a sensitivity, specificity, and accuracy of 61.5, 81.5, and 73.1%, respectively, in separating these two common benign lesions. Reports in the literature about the sensitivity of US to identify PA range from 55 to 82% and specificity ranges from 73 to 86% [14, 18]. PA are typically described as polycyclic or lobular tumors. Histologically, they consist of epithelial and myoepithelial cells and rarely undergo internal cystic degeneration [5]. Thus, on US, we expect PA to present as irregular tumors with well-defined borders, slightly heterogenous, and usually with no or poor vascularization. On the other hand, MA are described as oval and consist of mixed solid and cystic components, so should appear on US as well-defined, hypoechoic, heterogenous tumors with moderate or high vascularization [12]. However, PA can have cystic changes and hemorrhages within the tumor, especially in larger masses [5]. In addition, similarly, MA can present atypically as slightly heterogenous tumors similar to PA. The sonomorphologic characteristic of both these tumors surely depends on the proportion of their components: epithelial and myoepithelial cells in PA and cystic and solid tissues in MA [5]. In our study, we identified hypoechogenicity, vascularization, and enlargement of intraparotid lymph nodes as the most specific features in US differential diagnosis of PA and MA. Manosour et al. suggested that vascular pattern and acoustic enhancement are much more significant than margin definition, shape, and echogenicity [8]. The increased acoustic enhancement deep to the tumor on US is connected with cystic tumor changes that also appear as hypoechoic areas inside the mass. Thus, increased acoustic enhancement reflects the hypoechogenicity of the tumor. According to the study of Shmizu et al., parotid lesions with multiple anechoic areas were with very high sensitivity MA, whereas lobular shape and rather homogeneous echotexture predicted PA [9]. Zajkowski et al. confirmed that the hypoechoic areas are much more specific for MA than PA; however, there was no significant difference of the tumor’s shape between MA and PA in his study [18].

Recently, the role of contrast-enhanced ultrasound (CEUS) in the assessment of salivary gland tumors has been raised in the discussion. Using this method, a more detailed evaluation of the vascular pattern within the lesion is possible. In addition, numerical coefficients proposed for this method allow for a measurable comparison of results. David et al. presented the review of the literature concerning CEUS in parotid gland lesions [19]. In most of reviewed studies, the authors confirmed the high accuracy of CEUS in the diagnosis of malignant lesions and in the differentiation between PA and MA [19]. However, the major limitation of presented studies was limited population of patients. Mansour et al. in 2017 proposed a multimodal ultrasonographic pathway, including high-resolution ultrasound, color Doppler, CEUS, and elastography as the most efficient in increasing the specificity and positive predictive value in evaluation of parotid gland lesions [20].

## Conclusions

Our study confirms that high-resolution US with color Doppler can identify a variety of factors of some diagnostic utility in assessing parotid gland lesions. Echoinhomogenicity and increased vascularity were proven as the most reliable features for defining the malignant character of a lesion. We identified hypoechogenicity, vascularization, and enlargement of intraparotid lymph nodes as the most specific features in ultrasound differentiation of PA from MA. Unfortunately the diagnostic accuracy of US in distinguishing malignant from benign tumors and PA from MA does not reach a satisfactory diagnostic level. US has gained widespread acceptance as an economical, safe, and useful method for detecting and assessing parotid gland masses. In experienced hands, these studies can yield important diagnostic clues. However, this information should still be supplemented with fine needle aspiration biopsy to obtain the highest preoperative diagnostic accuracy.

## References

[1] Hugh CD, Myers E, Ferris RL (2007). Imaging of salivary gland. Salivary gland disorders.

[2] Harish K (2004). Management of primary malignant epithelial parotid tumors. Surg Oncol.

[3] Carlson ER, Ord R (2008) Tumors of the Parotid Gland. In: Carlson ER, Ord R (eds) Textbook and color atlas of salivary gland pathology. Wiley-Blackwell, Iowa, pp 171–198

[4] Wong DS (2001). Signs and symptoms of malignant parotid tumours: an objective assessment. J R Coll Surg Edinb.

[5] Lee YYP, Wong KT, King AD (2008). Imaging of salivary gland tumors. Eur J Radiol.

[6] Bozzato A, Zenk J, Greess H, Hornung J, Gottwald F, Rabe C, Iro H (2007). Potential of ultrasound diagnosis for parotid tumors: analysis of qualitative and quantitative parameters. Otolaryngol Head Neck Surg.

[7] Izzo L, Sassayannis PG, Frati R (2004). The role of echo colour/power Doppler and magnetic resonance in expansive parotid lesions. J exp Clin Cancer Res.

[8] Mansour N, Stock KF, Chaker A, Bas M, Knopf A (2012). Evaluation of parotid gland lesions with standard ultrasound, color duplex sonography, sonoelastography and acoustic radiation force impulse imaging: a pilot study. Ultraschall Med.

[9] Shimizu M, Ussmuller J, Hartwein J (1999). Statistical study for sonographic differential diagnosis of tumorous lesions in the parotid gland. Oral Surg Oral Med Oral Pathol Oral Radiol Endod.

[10] Mittal S, Vinayak V, Grover Kumar M (2013). Imaging criteria for salivary gland tumors-an overview. Ind J Contemp Dent.

[11] Haels J, Lenarz T (1986). Ultrasound diagnosis of benign and malignant parotid tumors. Laryngol Rhinol Otol.

[12] Shimizu M, Ussmuller J, Hartwein J (1999). Statistical study for sonographic differential diagnosis of tumorous lesions in the parotid gland. Oral Surg Oral Med Oral Pathol Oral Radiol Endod.

[13] Goto TK, Yoshiura K, Nakayama E (2001). The combined use of US and MR imaging for the diagnosis of masses in the parotid region. Acta Radiol.

[14] Bialek EJ, Jakubowski W, Karpinksa G (2003). Ultrasonography in diagnosis and differentiation of pleomorphic adenomas: work in progress. Arch Otolaryngol Head Neck Surg.

[15] Mansour N, Bas M, Stock KF, Strassen U, Hofauer B, Knopf A (2015). Multimodal ultrasonographic pathway of parotid gland lesions. Ultraschall Med.

[16] Hausegger KW, Krasa H, Pelzman W (1993). Sonography of the salivary glands. Ultraschall Med.

[17] Schick S, Steiner E, Gahleitner A (1998). Differentiation of benign and malignant tumors of the parotid gland: value of pulsed Doppler and color Doppler sonography. Eur Radiol.

[18] Zajkowski P, Jakubowski W, Bialek EJ (2000). Pleomorphic adenoma and adenolymphoma in ultrasonography. Eur J of Ultrasound.

[19] David E, Cantisani V, De Vincentiis M, Sidhu PS, Greco A, Tombolini M, Drudi FM, Messineo D, Gigli S, Rubini A, Fresilli D, Ferrari D, Flammia F, D’Ambrosio F (2016). Contrast-enhanced ultrasound in the evaluation of parotid gland lesions: an update of the literature. Ultrasound.

[20] Mansour N, Bas M, Stock KF, Strassen U, Hofauer B, Knopf A (2017). Multimodal ultrasonographic pathway of parotid gland lesions. Ultraschall Med.

